# Usefulness of a Digitally Assisted Person-Centered Care Intervention: Qualitative Study of Patients’ and Nurses’ Experiences in a Long-term Perspective

**DOI:** 10.2196/46673

**Published:** 2023-05-18

**Authors:** Mette Linnet Olesen, Sine Rossen, Rikke Jørgensen, Line Langballe Udbjørg, Helena Hansson

**Affiliations:** 1 Department of Gynecology The Interdisciplinary Research Unit of Women’s, Children’s and Families’ Health Copenhagen University Hospital Rigshospitalet Copenhagen Denmark; 2 Copenhagen Centre for Cancer and Health Municipality of Copenhagen Copenhagen Denmark; 3 Unit for Psychiatric Research Department of Psychiatry Aalborg University Hospital Aalborg Denmark; 4 Department of Clinical Medicine Aalborg University Aalborg Denmark; 5 National Health Portal/Sundhed.dk Copenhagen Denmark; 6 Department of Paediatric and Adolescent Medicine Copenhagen University Hospital Rigshospitalet Copenhagen Denmark; 7 Department of Clinical Medicine University of Copenhagen Copenhagen Denmark

**Keywords:** digital technology, digital nursing, digitally assisted guided self-determination, empowerment, self-management, person-centered care, qualitative, service design, patient care, nurse, quality of life, interview, web-based questionnaire, functionality, support, training, implementation, self-determination, autonomy, agency, person centered, patient centered, client focus, gynecology, oncology, health knowledge, health care professional, health care provider, HCP, mobile phone

## Abstract

**Background:**

Person-centered care responsive to individual preferences, needs, and values is recognized as an important aspect of high-quality health care, and patient empowerment is increasingly viewed as a central core value of person-centered care. Web-based interventions aimed at empowerment report a beneficial effect on patient empowerment and physical activity; however, there is limited information available on barriers, facilitators, and user experiences. A recent review of the effect of digital self-management support tools suggests a beneficial effect on the quality of life in patients with cancer. On the basis of an overall philosophy of empowerment, guided self-determination is a person-centered intervention that uses preparatory reflection sheets to help achieve focused communication between patients and nurses. The intervention was adapted into a digital version called digitally assisted guided self-determination (DA-GSD) hosted by the Sundhed DK website that can be delivered face-to-face, via video, or by the combination of the 2 methods.

**Objective:**

We aimed to investigate the experiences of nurses, nurse managers, and patients of using DA-GSD in 2 oncology departments and 1 gynecology department over a 5-year implementation period from 2018 to 2022.

**Methods:**

This qualitative study was inspired by action research comprising the responses of 17 patients to an open-ended question on their experience of specific aspects of DA-GSD in a web questionnaire, 14 qualitative semistructured interviews with nurses and patients who initially completed the web questionnaire, and transcripts of meetings held between the researchers and nurses during the implementation of the intervention. The thematic analysis of all data was done using NVivo (QSR International).

**Results:**

The analysis generated 2 main themes and 7 subthemes that reflect conflicting perspectives and greater acceptability of the intervention among the nurses over time owing to better familiarity with the increasingly mature technology. The first theme was the different experiences and perspectives of nurses and patients concerning barriers to using DA-GSD and comprised 4 subthemes: conflicting perspectives on the ability of patients to engage with DA-GSD and how to provide it, conflicting perspectives on DA-GSD as a threat to the nurse-patient relationship, functionality of DA-GSD and available technical equipment, and data security. The other theme was what influenced the increased acceptability of DA-GSD among the nurses over time and comprised 3 subthemes: a re-evaluation of the nurse-patient relationship; improved functionality of DA-GSD; and supervision, experience, patient feedback, and a global pandemic.

**Conclusions:**

The nurses experienced more barriers to DA-GSD than the patients did. Acceptance of the intervention increased over time among the nurses in keeping with the intervention’s improved functionality, additional guidance, and positive experiences, combined with patients finding it useful. Our findings emphasize the importance of supporting and training nurses if new technologies are to be implemented successfully.

## Introduction

### Background

Person-centered care that is responsive to individual preferences, needs, and values is recognized as an important aspect of high-quality health care [1,2], and patient empowerment is increasingly viewed as an important core value of person-centered care [3,4]. To cope with the increasing burden of chronic diseases, enabling patient empowerment is considered essential for achieving high-quality health care systems in the future [4].

A 2013 systematic review investigating the effect of web-based interventions aimed at increasing patient empowerment and physical activity in chronic conditions assessed the relevance of the interventions for cancer survivors [5]. The authors concluded that web-based interventions beneficially affected patient empowerment and physical activity; however, limited information was provided on barriers, facilitators, and user experiences [5]. A 2021 review examining the effect of digital self-management support tools in patients with cancer found that they also seemed to have a beneficial effect on quality of life, whereas the effects on other outcomes were inconsistent [6].

Empowerment is the underlying philosophy of guided self-determination (GSD), in which the overall goal is to support patients’ development of life skills to improve their self-management of chronic conditions [7,8]. A randomized controlled trial testing the analog version of the GSD adapted for gynecologic cancer in a hospital setting invited 719 women to participate; 82 women declined because they thought the time they had to spend on transportation was excessive. Other reasons for declining participation were comorbidities and a lack of energy. Several participants who lived far away requested access to a digitally assisted version of the GSD to participate in the intervention [9]. Another study supports this finding, which indicates that technology-based approaches can be used to alleviate long travel times and provide better supportive care in rural districts [10]. Furthermore, research shows that important disparities exist in mental health outcomes between rural and urban cancer survivors [11].

To increase access to health care, digitalizing and incorporating the option of using video conversations in person-centered interventions is of great importance. From 2016 to 2017, the Danish National Health Portal (DNHP), Sundhed DK [12], developed the digitally assisted GSD (DA-GSD) intervention based on the analog version, but it has new functionalities and the option of using videoconferencing [13]. The intervention is described in further detail in the *Methods* section.

### Objective

DA-GSD was implemented in 2018 at 3 hospital departments in Denmark treating women with cancer and endometriosis. Documenting the maturation of the technology when switching from an analog to a digital version that uses not only face-to-face but also video conversations is important in terms of successfully developing future interventions. Other aspects that are important to examine and document include barriers and facilitators, user experience, and identification and fulfillment of the individual’s need for support in the digital environment. This paper reports on the implementation process with the aim of exploring the experiences of nurses, nurse managers, and patients with DA-GSD over a 5-year period from 2018 to 2022.

## Methods

### Design

Inspired by action research [14], this qualitative study involved the implementation of DA-GSD, in which the users were expected to acquire new skills and adjust their mindset, leading to the generation of important knowledge at the individual and organizational levels. Therefore, action research offers an ideal foundation for taking a reflective approach that provides a stepwise transformation of DA-GSD and progressive understanding in an iterative, cocreative process that focuses on reflection in relation to functionality, barriers, facilitators, user experience and a continuous adjustment to the individual needs of the patients and nurses.

### Setting

The study was conducted from 2018 to 2022 at 3 different departments at a university hospital in Copenhagen, Denmark, that were implementing DA-GSD: a gynecology department surgically treating patients with gynecological cancers as well as women with endometriosis as of 2020, an oncology department treating patients with various cancer diagnoses, and an oncology rehabilitation unit counseling patients with a variety of cancer diagnoses.

### DA-GSD Intervention

#### Overview

DA-GSD is based on the analog version of GSD [7,8], in which the main features are reflection sheets designed to help patients prepare before speaking with health care professionals (HCPs) trained in using GSD. The reflection sheets help patients clarify their values and initiate reflection on their needs and resources. The completed reflection sheets form the foundation for mutual person-centered counseling, with some of the sheets specifically designed to support shared decision-making. The HCPs are certified in GSD and advanced communication to facilitate person-centered dialogue and problem-solving. The analog version of GSD was originally developed for patients with diabetes but has since become a method used and evaluated for various other diseases and conditions [15].

DA-GSD was developed from 2016 to 2017 by the DNHP Sundhed DK website [12] in an agile process that involved actor and stakeholder analyses and collaboration between project managers, IT architects, and researchers [13].

#### Service Design in Health Care

Throughout the process of converting the traditional analog reflection sheets into a digital version for patients and HCPs, a service design methodology was used [12], which involves taking a holistic approach that prioritizes identifying and meeting user needs and optimizing their service journey [16].

The partnership established among the HCPs; patients; and cross-functional team of the Sundhed DK website [12], which comprised product managers, designers, user experience specialists, and software developers, was highly collaborative and productive. The main focus of the project was to ensure that the digital aspects did not compromise the existing GSD method but instead created a seamless and sophisticated solution that leveraged the capabilities of digital technology to empower patients and provide HCPs with a highly effective tool, while integrating it into the daily lives of users.

#### Log-in

HCPs used their secure work digital ID to access DA-GSD via the DNHP Sundhed DK website [12] to create specific sessions targeting individual patients and reflection sheets to be completed by the patient. After this step, the patients were asked to log in with their secure personal digital ID to fill out and save their sheets.

#### Analog Versus Digital Reflection Sheets

The content of the digital reflection sheets was the same as that of the analog sheets, but their functions had changed. Some digitalized sheets were interactive and responsive to mobile devices. One of the analog sheets asks the patients to draw a picture of their experience of living with their disease, whereas the corresponding digital version permits the users to upload images to illustrate their experience. Finally, the problem-solving analog sheets, which comprised 4 pages on feelings, observations, goals, and actions concerning a specific problem, were now completed digitally and could be condensed into 1 page summarizing all 4 aspects to support problem-solving.

#### DA-GSD Provided Face-to-face, via Video, or a Combination

**Figure 1:**
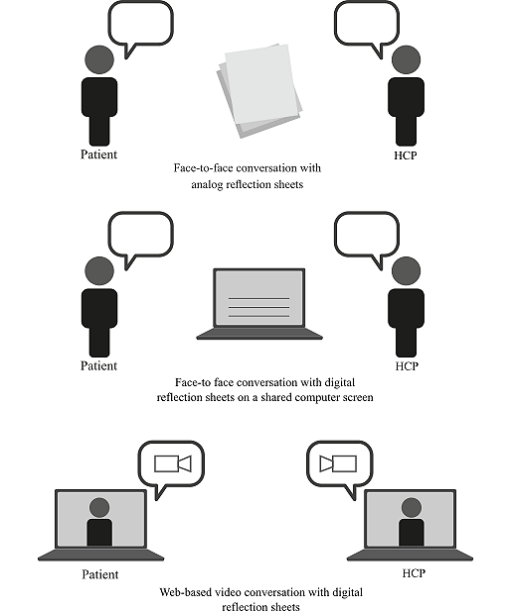
Digitally assisted guided self-determination provided face-to-face, via video, or as a combination of the 2. HCP: health care professional.

DA-GSD allowed nurses and patients to collaborate on the digital sheets in a face-to-face meeting to discuss them on a shared computer screen, via videoconference and screen sharing, or by using a combination of the 2 approaches (Figure 1).

**Figure 2:**
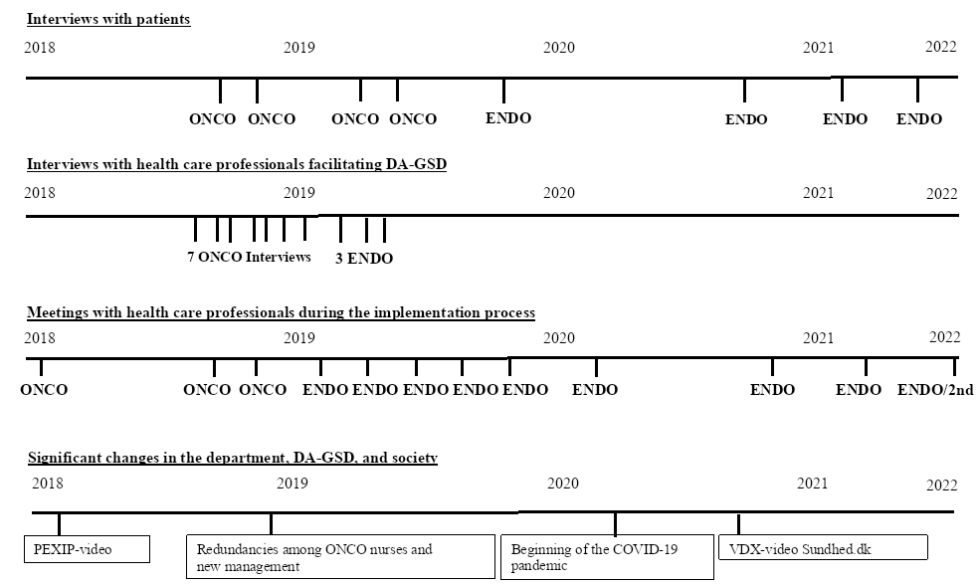
Timeline. DA-GSD: digitally assisted guided self-determination; ENDO: endometriosis; ONCO: oncological disease.

Pexip (Figure 2), which was the first video feature developed, involved a cumbersome process requiring user information for patients and nurses to initiate a video meeting. The nurses had to copy and paste guest codes to send to patients and themselves, which had to be typed on the day of the video conversation because codes generated by Pexip were frequently identified as spam. Another disadvantage of Pexip was that the image visible to the patients was small when they shared their digital sheets on the screen.

In the autumn 2020, video consultation using VDX was introduced [12], which is a public, cross-sector video infrastructure commonly used by hospitals across Denmark and is adapted to PCs and mobile devices. To plan a meeting, nurses must copy and paste a link to the web-based meeting room they have created and send it to the patient with a specific date and time for the meeting. Nurses and patients use their secure digital IDs to log into the web-based meeting room, where full-scale digital reflection sheets can be shared.

### Population and Inclusion Criteria

There were different inclusion criteria for nurses with managerial responsibility and the other nurses. Inclusion criteria for the former were: managerial responsibility for staff who were implementing DA-GSD, and not necessarily possessing certification in GSD or DA-GSD, or in facilitating them. Inclusion criteria for the latter were: being certified in GSD [17] and in facilitating DA-GSD. The inclusion criteria for patients were as follows: they must be aged >18 years; be able to understand, read, and write Danish; have a cancer or endometriosis diagnosis; and participate in DA-GSD via face-to-face conversation, videoconference, or a combination of the 2.

A total of 10 nurses with a median range of 40 to 49 years of age with 4 to 37 years (median 26.5 years) of nursing experience participated from the 3 departments. Of the 10 nurses, 5 (50%) nurses had a master’s degree and 3 (30%) had managerial responsibility, 1 of whom facilitated GSD, whereas the remaining 2 nurses facilitated the implementation of GSD in their department. Nurses had generally conducted 10 GSD conversations on average, but the number ranged from 5 to >100, as shown in Table 1, which presents the patient characteristics.

**Table 1:** Characteristics of participating patients (N=17).

Number or ID		Age (years)	Relationship	Education	DA-GSD^a^ conversations	Video conversation	Interviewed
**Patients with an oncological disease (N=6)**							
	1	45-49	In a relationship	≥1 shorter course	4	N/A^b^	✓
	2	50-54	Single	3-4 years of higher education	4	4	✓
	3	>60	In a relationship	≥1 shorter course	4	2	✓
	4	55-59	In a relationship	3-4 years of higher education	5	N/A	✓
	5	50-54	In a relationship	Vocational education or skilled worker	5	N/A	N/A
	6	>60	Single	>4 years of higher education	4	N/A	N/A
**Patients with endometriosis (N=11)**							
	1	40-44	In a relationship	Vocational education or skilled worker	3	N/A	✓
	2	<30	In a relationship	3-4 years of higher education	3	2	✓
	3	30-34	In a relationship	3-4 years of higher education	4	N/A	✓
	4	40-44	In a relationship	Vocational education or skilled worker	3	3	✓
	5	>45	In a relationship	3-4 years of higher education	3	N/A	N/A
	6	35-39	In a relationship	None	4	N/A	N/A
	7	35-39	In a relationship	Vocational education or skilled worker	3	N/A	N/A
	8	30-34	Single	Vocational education or skilled worker	2	N/A	N/A
	9	>45	In a relationship	3-4 years of higher education	4	N/A	N/A
	10	35-39	Single	3-4 years of higher education	3	N/A	N/A
	11	35-39	Single	Vocational education or skilled worker	3	3	N/A

^a^DA-GSD: digitally assisted guided self-determination.

^b^N/A: not applicable.

### Recruitment and Data Collection

Nurses and patients were recruited between 2018 and 2022. The participating nurses and nurse managers were recruited by the first author (MLO). The patients were included continuously by the participating GSD nurses. We did not register patients who declined to participate in the study.

Semistructured interviews with 10 nurses were conducted during the initial implementation of DA-GSD. One nurse who participated throughout the entire study was interviewed twice, once at the beginning and again in 2022. The interview guide for nurses focused on their experience of using DA-GSD and covered functionality, the perceived need for DA-GSD, favorability of the surrounding circumstances, barriers, challenges, need for support, their role in facilitating DA-GSD, and data security. During the 5-year implementation period, MLO and a project assistant held regular implementation meetings with the nurses regarding their current experiences and challenges. Interviews and meetings were audio recorded, transcribed, and included as data. Knowledge from these meetings, specifically regarding the functionality of DA-GSD, was continuously reported back to Sundhed DK website [12] to provide a foundation for adjusting the intervention.

The included patients reported their sociodemographic details before their first DA-GSD conversation. After the last DA-GSD conversation, all patients were asked to complete a web-based questionnaire that contained an open-ended question about their overall experience with the digital reflection sheets. These answers were included in the data. Individual semistructured interviews were conducted with a convenience sample [18] comprising 8 patients who had finished participating in the DA-GSD (Table 1). The patient interview guide focused on their experience with DA-GSD, including functionality, what worked well or poorly, the need for support, how they felt about using technology for disease management, and how they experienced their relationship with the nurse during DA-GSD. The interviews were audio recorded and transcribed. Figure 2 provides an overview of the overall data collection process.

### Ethical Considerations

All participants received oral and written information and were given time to consider their participation before providing written informed consent. The study was conducted in accordance with the Declaration of Helsinki and was registered with the Danish Data Protection Agency (file RH-2017-248, I-Suite 05720). Ethics approval was not legally required.

### Data Analysis

The data were analyzed thematically by following a 5-step process [19]. First, the authors read the transcriptions of the interviews, meetings, and questionnaire responses. Second, preliminary codes were noted in the margins during the reading. Next, the codes were discussed in meetings that involved searching for themes, which resulted in meaningfully collecting the codes into 4 preliminary themes covering various perspectives on the ability of patients to engage in DA-GSD, the nurse-patient relationship, DA-GSD’s functionality, and data security. Upon reviewing these themes, all authors agreed that they could be categorized as subthemes under the overarching theme of nurses and patients having different perspectives on the barriers to DA-GSD. Other data reflected a change in nurses’ attitudes toward DA-GSD over time, with the ongoing analysis generating an additional theme regarding this change and subthemes describing what influenced the change. Fourth, the themes and subthemes were reviewed and checked in relation to the coded data and overall data set. Finally, the themes were named and defined.

## Results

### Demographic Characteristics

A total of 10 nurses and 17 patients were included from 2018 to 2022. Of the 17 patients, 12 (71%) patients received DA-GSD via face-to-face conversations, 3 (18%) via video only, and 2 (12%) via a combination of face-to-face and video conversations (Table 1).

### Themes

#### Overview

There were 2 main themes, one on conflicting perspectives that contained 4 subthemes and the other on the development of DA-GSD over time that comprised 3 subthemes (Textbox 1).

**Themes and subthemes.**

Different experiences and perspectives of nurses and patients concerning barriers to using digitally assisted guided self-determination (DA-GSD)

- Conflicting perspectives on the ability of patients to engage with DA-GSD and how to provide it
- Conflicting perspectives on DA-GSD as a threat to the nurse-patient relationship
- Functionality of DA-GSD and available technical equipment
- Data security

What influenced the increased acceptability of DA-GSD among the nurses over time

- Re-evaluation of the nurse-patient relationship
- Improved functionality of DA-GSD
- Supervision, experience, patient feedback, and a global pandemic

#### Different Experiences and Perspectives of Nurses and Patients Concerning Barriers to Using DA-GSD

This theme comprises 4 subthemes describing aspects that some users experienced as barriers to using DA-GSD and about which they, in several cases, had opposing perspectives. Many of the barriers that most nurses described were not reflected in the patients’ experiences.

##### Conflicting Perspectives on the Ability of Patients to Engage With DA-GSD and How to Provide It

In general, the nurses were positive about technology and GSD being digitalized, but they had many deliberations about the patients’ prerequisites for participating in DA-GSD, especially during the beginning of the implementation process. This involved, for example, concerns about age, digital skills, cognitive function, fatigue, and the level of digital literacy. They appeared to protect the patients from the challenges they expected to arise from DA-GSD. Only 2 nurses experienced DA-GSD as easy to use from the outset, and they were younger or positively motivated by the unpredictability of their work tasks:

I think it’s super exciting and it’s modern. And that’s how it’s going to be; it’s part of technological development that we also start doing things like this...and that everything shouldn’t be in paper form, and it’s clearly aimed at a target group that is younger and thus comfortable with all this...I have a concern that it may exclude someone who does not have strong [digital] literacy because they’re not safe inside that world.

Nurse 3

As expected by the nurses in the beginning, many patients said that DA-GSD was challenging in terms of their memory, fatigue, and sometimes the length of the reflection sheets. Furthermore, using the save feature (a flashing heart in the middle of the screen) was a lengthy and challenging process that made filling out the digital sheets difficult, in addition to the fact that they did not always work as intended, and the video feature had a complicated log-in process. Nevertheless, they did not mind overall and were generally highly positive about DA-GSD throughout the whole process, and they felt that the intervention fulfilled an unmet need.

During the implementation process, nurses’ assumptions or understanding of patients’ digital skills were sometimes not in agreement with patients’ actual experiences and skill levels. In one case, a nurse described how she included a patient who she felt did not have the skills to participate:

I felt that I...twisted the arm of someone [patient] who was not very digitally literate and who chose to say, well, I want that, because I want these conversations with you [the nurse] so much...That was a big challenge I just gave her.

Nurse 3

This did not fully align with the patient’s experience, which the patient described as not only challenging but also good and successful:

I hadn’t visited Sundhed.dk [DNHP] before...but I got in and found all the sheets. I was so happy. Because my memory does not work as well as it should, I find it difficult to remember.

Patient 1, oncology

Other patients said the following about DA-GSD: “works great,” “I could easily figure it out,” and “I’m used to logging in and checking test answers and stuff like that.” In general, their descriptions of the benefits overshone the challenges they experienced. One woman was especially excited about digitalized sheets that facilitated the problem-solving process:

It was visual somehow, wasn’t it...? It was pretty good...I remember thinking while I was answering the questions, I thought: “It can’t be connected. It just doesn’t add up”...and then it was presented on that page [where reflections on feelings, observations, goals, and actions about a problem are summarized on a final sheet]...“It does add up! ”, it’s like a SWOT [strengths, weaknesses, opportunities, and threats] analysis, in reality.

Patient 3, oncology

Initially, the Pexip video feature was somewhat cumbersome for nurses and patients. Moreover, because they felt responsible for the technical functionality of DA-GSD, including the video feature, they were concerned when the patients requested video conversations:

As a professional, I’m a little bit worried about the technology...in terms of whether it causes any problems and whether it can...make it difficult to have a conversation.

Nurse 2

A pattern emerged throughout the implementation period that involved the nurses trying to screen the patients’ digital skills to ensure that they could manage the challenges involved in using DA-GSD:

It has to be a patient who really understands IT and is not afraid of it and who is completely ready for it...

Nurse 7

Although the patients described difficulties in getting the video feature to work, being challenged was not the dominant narrative that emerged when they were interviewed regarding the video conversations. Their focus was primarily on the benefits of the conversation:

I felt like I really got something out of it when we finished the hour-long videoconference, and I had some tasks to work on for the next time a fortnight later. And I actually looked forward to having that conversation again, because there were some things I was looking forward to talking about.

Patient 2, oncology

Importantly, this quote is from the time when the intervention had just begun and Pexip provided the first video feature, which was even more difficult to install for this patient as she used an Apple computer. Nevertheless, the patient described using the video feature as crucial to her because she would otherwise have been unable to participate:

If I had to show up [in person], I wouldn’t have come.

Patient 2, oncology

She lived far away and did not have the strength or money to pay for a long trip.

##### Conflicting Perspectives on DA-GSD as a Threat to the Nurse-Patient Relationship

Initially, during the commencement of the implementation, most nurses were concerned that DA-GSD would negatively affect or be a threat to their relationship with the patients. This sense of uneasiness was reflected in both face-to-face conversations using digital sheets on a shared PC and video conversations that involved collaborating on the sheets with screen sharing:

The thing about constantly having to look at a screen [face-to-face conversations with reflection sheets on a shared screen]. I really like making eye contact with people. And there’s also something about keeping an eye on reactions and (...). You can get a little preoccupied with a screen like that, so (...) maybe I could also see such a barrier in relation to the conversation itself, how...so it might be a slightly different conversation. I don’t know, it’s not certain.

Nurse 3

In relation to the video conversations, their considerations focused on their ability to comfort the patients:

If you have to do that video...that slightly close contact you have with the patient when you normally sit next to each other and can hand them a handkerchief and bring them a glass of water...you don’t have that option long distance, so it could well be a bit negative to have them further away.

Nurse manager 8

Although the nurses with managerial responsibilities also hesitated about the quality of the relationship via video conversations, all the nurses (including those without the managerial responsibilities) recognized that video conversations were better than no access to health care for patients living far away:

Well, I think I have a foot in two camps because..., for me, communication is when you sit and have eye contact, and you can sense each other, and you sit across from each other, and you’re just people sitting and having a conversation. And I know that well, we can’t keep doing that because patients, especially here with us, come from far away [Faroe Islands, Greenland, and Jutland], and I think it would be a shame if those patients were prevented from having conversations because of the distance is a barrier.

Nurse manager 5

The nurses shared feedback during the regular implementation meetings. They discussed patients who wanted to do video conversations, and if they agreed that it was a good idea, they would recommend that the first conversation take place face-to-face at the hospital to help establish the relationship. This approach aligned with patients who combined face-to-face and video conversations. The nurses felt that the initial face-to-face conversation added value and believed that it was the reason video conversations were experienced positively subsequently.

In general, the patients experienced no deterioration in their relationship with the nurses when using DA-GSD with both types of conversations. By contrast, some saw video conversations as an advantage:

...when you’re sitting in the comfort of your own home, you can open up a little more than if you sat and were met head-on. I’ve built up a...a minor phobia of doctors...and then you can say that this [video conversation] is way better because there’s that kind of barrier somehow (...) I have to say, so I think it has worked.

Patient 2, endometriosis

##### Functionality of DA-GSD and Available Technical Equipment

During the data analysis, it became clear that many of the initial challenges patients experienced early in the study and the ones the nurses discussed at the implementation meetings disappeared over time, that is, patients did not mention them later in the implementation process. One possible reason for this is that DA-GSD and the video features were continuously adjusted during the study period based on feedback from the intervention [12], accelerating the maturation and improvement of the technology.

Patients included early in the study found that the save feature in the digital sheets, a flashing heart in the middle of the screen, was an annoying hindrance “...and then suddenly that heart appeared, almost as if it had to update something” (Patient 3, oncology). This issue continued for quite some time:

Sometimes, if you wrote it quickly, it couldn’t keep up...then the heart blinked...in the end, I gave up and wrote everything down by hand.

Patient 4, oncology

Early in the study, the complicated setup of the Pexip video feature and its instability were perceived as challenges:

...how do we do it now [use video]...and it never worked out and then suddenly it crashed when I finally had to having a video conversation. The system didn’t work...

Nurse 7

Neither the nurses nor the patients described having trouble logging in to the DNHP on Sundhed DK website [12] with their digital IDs to initiate their DA-GSD, although some had difficulty retrieving their digital reflection sheets because they forgot the name of the intervention.

In addition to the challenges concerning functionality, the nurses lacked the technical equipment needed to conduct video conversations, making them even more skeptical about video conversations:

Well, yes...[pause], secondly, it’s at least important that all departments have a computer with a camera. And the desktop computers don’t have that, at least the way things are in the department right now. I’ve been thinking about it lately...In any case, we must have access to a computer equipped with a camera...

Nurse 2

##### Data Security

Almost all patients felt completely safe about having sensitive data hosted at DNHP Sundhed DK website [12]:

I feel very comfortable with that. I was a little surprised that it [this question] was asked at all.

Patient 3, endometriosis

I think there’s so much other data about me that is digital.

Patient 2, endometriosis

There were only a few who had thought about whether unauthorized individuals could gain access to and see data or whether data could be leaked:

Actually, I feel completely unworried, and yet, I know very well that everything can be leaked in the end. It’s not because I have something to hide, but whether I fully trust that it won’t get leaked one day, I don’t know if I do...

Patient 4, oncology

This was somewhat in contrast to what most nurses felt, who did not consider data security an issue when using the DNHP, Sundhed DK website [12]:

I don’t think that’s a problem, we already have data there, right?

Nurse 9

Several patients wanted other HCPs besides nurses to be able to see their data and the challenges they were collaborating on with the nurses during their DA-GSD conversations:

Others should be able to see the data so that you don’t have to explain yourself so many times...that they [the completed reflection sheets] would become part of your medical record, perhaps...so the doctor can also see how the person is actually doing.

Patient 1, endometriosis

Both patients with endometriosis and cancer thought that data of this nature should be accessible to the multidisciplinary team surrounding the patient:

I want the doctors to be able to see it, so they can see how I feel!

Patient 1, oncology

#### What Influenced the Increased Acceptability of DA-GSD Among the Nurses Over Time

##### Re-evaluation of the Nurse-Patient Relationship

As the nurses gained more experience using DA-GSD, their concerns about whether the nurse-patient relationship might deteriorate disappeared, regardless of how the intervention was provided. The following quote is about face-to-face meetings, which include sharing digital reflection sheets:

It’s going really well. During the conversation, we sit opposite each other and occasionally look at the screen. I don’t see it as a problem that the computer screen becomes part of the conversation. I don’t see it affecting the relationship between me and the patient

Nurse at endometriosis implementation meeting 2019

One nurse who had used DA-GSD continuously throughout the implementation process was interviewed twice, once at the beginning and once toward the end of the implementation period. Her experiences also illustrated the general increase in recognizing that the relationship remained intact. Despite her concerns regarding the nurse-patient relationship at the beginning of the study, using videos for DA-GSD became a tool that she offered to every patient by the end of the study period. At that point, she did not worry about or even consider its effect on forming a relationship and stated the following at her follow-up interview:

I actually had someone [patient] the other day who I had to book [for DA-GSD], and then I asked what she preferred...she was from XXX [less than 20 kilometers away], but she wanted video.

Nurse manager 8

A common pattern was that the nurses saw a trusting relationship developing with patients when they used DA-GSD:

You get closer to the patient, I think...and when I meet her, it’s a big hug in the hallway and she feels very attached to me.

Nurse 7

##### Improved Functionality of DA-GSD

Over time, the nurses experienced that the functionality of DA-GSD matured, for example, the annoying blinking heart that served as the save feature completely disappeared:

After Sundhed.dk ran DA-GSD again, they [the patients] don’t find that it [DA-GSD] freezes anymore. It’s not something that the patients complain about.

Nurse at endometriosis implementation meeting 2019

The improved functionality coincided with the nurses describing that they were increasingly unconcerned about whether patients requested the analog version or DA-GSD:

Now I have tried both the digital one and what was printed out [analog GSD], and I think that the digital one just works really well. It’s easy for the patients to fill in and I don’t have to think about whether I have to print something out...it’s easy to log in to Sundhed.dk and...well, I’m comfortable with that. I actually don’t think of it as being anything special at all.

Nurse 10

The final video feature at DNHP Sundhed DK website [12] also appeared to run seamlessly:

We had to try it once to see if it worked [video conversations]...it worked. We continued with it...and it worked fine.

Patient 2, endometriosis

At the end of the implementation process, the digital reflection sheets were prioritized over the analog ones, and the nurses viewed video conversations as an excellent supplementary resource to offer patients whom they would otherwise have been unable to reach and support owing to long distances:

It’s a great extension of the method, isn’t it?...and also a resource, I think. Another tool we can have on the shelf, which might serve...? People who live far away.

Nurse 9

Notably, the statements supporting the mature, well-functioning version of the DA-GSD were made by patients and nurses who used it toward the end of the implementation process.

##### Supervision, Experience, Patient Feedback, and a Global Pandemic

At the beginning of the implementation process, the nurses had various preferences about how they would like to be taught about DA-GSD: “...for me it would be really nice with a little summary [of DA-GSD]” (nurse 7). Although written instructions were sufficient for some nurses, others wanted one of the researchers to join them in the beginning. Written instructions were drawn up and adjusted on an ongoing basis, and individual needs for instruction were met as much as possible. In addition, guidelines for the video conversations were prepared jointly with the intervention partners who provided the video features. In addition, tips and tricks that were presented at meetings were drawn up in writing and covered issues such as those concerning the physical and confidential setting of a video conversation, location in relation to the camera, and the use of headsets to optimize the sound. During the meetings inspired by action research, the nurses gave one another feedback and shared experiences about the challenges they encountered and how they approached them.

During the implementation process, the nurses became more comfortable with DA-GSD, owing to its improved functionality, ongoing instruction, the support provided by the researchers, and an increase in confidence arising from their own experiences and those of their colleagues. On the basis of their own experiences, they began to emphasize the advantages of the digital version over the analog version. Several nurses emphasized the advantage of being able to safely store reflection sheets on the web so that it could be easily retrieved by all parties in relevant contexts before and after the conversations:

The electronic GSD is perceived as much better than the analogue one. Patients won’t lose the sheets, and you can easily find what you’ve talked about. As an HCP, you can easily look at the sheets again, if necessary.

Endometriosis implementation meeting 2019

As described earlier, the nurses felt technically challenged, especially at the beginning of the implementation process. They felt professional responsibility for the DA-GSD’s functionality when offering it to patients. Their evaluation of DA-GSD was positively affected by patients who apparently embraced the intervention and described it as useful and supportive, despite technical challenges:

It’s the best sessions I’ve ever attended, and I’ve attended a heck of a lot.

Patient 4, oncology

Some of the positive benefits of DA-GSD that the patients described were that they were being seen as a whole person, as it enabled them to exchange knowledge with an HCP familiar with their disease and receive support for successful self-management.

Positive patient feedback about DA-GSD video conversations also appeared to influence the nurses’ attitudes toward the use of video:

I think it worked quite well and, for me, it’s been a good opportunity to do it digitally.

Patient 2, endometriosis

I have tested that [DA-GSD] with some patients...they liked it...they think it made sense...now it’s young women I’ve had, and they use digital tools—without generalizing—very easily, yes. So, exactly for that patient group, because they are young women, I think it works fine. At least those are the ones I’ve done it with.

Nurse 10

In March 2020, the COVID-19 pandemic changed the delivery of health care worldwide and also affected how the nurses experienced the usefulness of DA-GSD using video instead of face-to-face conversations:

An interesting observation was that, during video conversations, they saw the whole patient and could see the subtleties of their body language. When they meet face-to-face, only the eyes are visible [owing to the use of masks during the COVID-19 pandemic].

Report from endometriosis implementation meeting 2020

For other patients, using personal protective equipment during the pandemic meant that a video conversation was preferable to meeting face-to-face. Interestingly, they focused primarily on interpreting the nonverbal language:

I actually almost think that, with this corona, it’s been easier over video because you can see each other, instead of sitting with masks and shields and all that, so in that way, it’s actually been better.

Patient 4, endometriosis

The nurses agreed that the pandemic quickly made collaboration via video more common among both patients and HCPs. The pandemic also resulted in the provision of additional technical equipment for video conversations in departments:

In relation to facilities for video conversations in the outpatient clinic, there are also some new resources that were not there before the pandemic, for instance, video and computers that allow you to facilitate video conversations...We have three computers that are equipped with cameras...and then I found out that this, a portable camera that you can take with you, also works.

Follow-up interview, nurse manager 8

## Discussion

### Principal Findings

This study aimed to investigate the experiences of nurses, nurse managers, and patients with DA-GSD in a long-term implementation process. Our results show that nurses and nurse managers were more concerned about using DA-GSD and the various ways of using it than the patients. The nurses’ doubts regarding the ability of patients to use DA-GSD, as well as their concerns about it having a negative impact on the nurse-patient relationship, were baseless. Although DA-GSD did not always work optimally, the patients embraced the intervention and found it useful and rewarding. The improved functionality, nurses’ own experience with using DA-GSD, and patients’ positive feedback seemed to affect the acceptability of DA-GSD over time among the nurses. Of note, the global pandemic required rapid implementation of video consultations in health care worldwide, which likely also played an important role in its acceptability. In contrast to the nurses, only a few patients had concerns about data security.

### Discussion of Principal Findings

#### Ability of Patients to Engage With DA-GSD

The nurses and patients had conflicting perspectives on the ability of patients to engage with DA-GSD. Our study showed that the nurses’ concerns about patient characteristics that would be a barrier to using DA-GSD and would prevent them from benefiting from the intervention were unfounded, although other studies have found that they were warranted [20]. A previous study reported that cognitive impairment, older age, and difficulties in coping with technology are barriers to using telehealth [20], whereas a systematic review found that these factors, with the exception of cognitive impairment, are also patient-related barriers to adopting telemedicine [21]*.*

The nurses in our study felt responsible for the functionality of the technology and ensuring that the individual patients had the necessary digital skills before engaging in DA-GSD. A scoping review on the role of nurses and the skills needed to master technology and digital solutions [22] found that communication skills, adaptiveness, and problem-solving were needed to adapt the interaction to the patients’ digital skills and digital knowledge. Therefore, training nurses in new communication and technological skills was important for advancing their readiness to adopt telemedicine [22]. This is in line with our findings, which showed that the nurses felt responsible for the technical functionality and needed individual supervision in DA-GSD, and is reflected in feedback about the challenges they experienced to increase their confidence in facilitating DA-GSD.

In our study, the patients embraced DA-GSD and found it useful, which is in line with a review of web-based psychosocial interventions for cancer survivors [23] that found that most studies reported that patients welcomed web-based interventions positively.

#### The Nurse-Patient Relationship

Nurses’ expectations and concerns about DA-GSD affecting the nurse-patient relationship were a central aspect in our findings. According to the person-centered practice framework, the relationship is important [24] and the development of nurses’ relationship skills is a prerequisite for person-centered care that warrants the nurse’s ability to communicate on different levels using verbal and nonverbal communication [24]. One of the person-centered care processes that the framework describes is that nurses must be sympathetically present and recognize the uniqueness and value of the person by appropriately responding to cues [24]. The nurses in our study described initial concerns regarding their ability to communicate adequately being challenged because of impaired nonverbal cues and thus an inability to respond properly to them. Our findings are in line with a systematic review of qualitative studies examining nurses’ experiences as facilitators of telehealth applications [20]. Similar to our study, their findings show that nurses experienced a dilemma regarding the nurse-patient relationship when using telehealth; on the one hand, they reported a negative and changed relationship as a barrier and, on the other, an improved nurse-patient relationship as a facilitator [20]. The barriers in the review included loss of human contact [20], which was also the case in our study, although video conversation was only used in a small percentage of DA-GSD sessions, whereas another barrier was difficulty in getting to know the patient, which was not described as a barrier in our findings. This was perhaps the case because DA-GSD comprised the use of reflection sheets filled out in advance before sessions with an HCP and the systematic sharing of person-specific knowledge [15]. The review mainly identified positive experiences with using telehealth [20], which is in line with our findings, wherein most barriers disappeared when the nurses had positive experiences with DA-GSD. A study examining the use of video consultations by doctors also reported the fear of deterioration in the physician-patient relationship, for example, not shaking the patient’s hand and having eye contact prevented access to a great deal of important information that the doctors were reluctant to miss [25]. However, they found that they could maintain good contact and assess patients satisfactorily while achieving more relaxed interaction in video consultations [25]. This is completely in line with our findings, which showed that the relationship remained good and that some patients felt more secure doing video calls at home.

#### Functionality of DA-GSD and Lack of Technical Equipment

Our study found that 2 barriers to implementation were DA-GSD’s functionality and a lack of technical equipment, which is in line with a systematic review of the barriers and facilitators of eHealth services [26] that identified three categories of barriers: (1) individual*,* such as motivation, accessibility, and trust; (2) environmental and organizational*,* such as financial, political, and organizational structures; and (3) technical, such as services and design that do not suit user needs. These findings are in accordance with ours in which the nurses lacked motivation to initiate video conversations in the beginning because the initial Pexip video feature did not meet their needs for functionality, turning its functionality into a personal and technical barrier. Interestingly, the patients did not experience the technical barriers to the same degree as the nurses, perhaps because the benefits of DA-GSD outweighed the technical challenges and the nurses felt a great deal of professional responsibility toward its functionality. A study on patient rounds with relatives consulted on video also found that the nurses felt a huge responsibility for the technical setup and organizing it [27]. Other studies reported that HCPs resisted using telemedicine because it represented a threat to their clinical work [21,27]. Organizational barriers became visible in our study during the COVID-19 pandemic, which accelerated organizational readiness for the use of video consultations and, specifically, the purchase of additional technical equipment. Other barriers to adopting telemedicine are its cost and outdated equipment [21].

### Comparison With Prior eHealth Intervention Based on the GSD Studies

GSD was previously digitalized in an eHealth intervention targeting patients with type 2 diabetes in Norway [28-31] and as a web-based GSD program in Australia [32,33]. The Norwegian version, eHealth intervention based on the GSD [28-30], was used in a general practice in 4 e-consultations. After the initial face-to-face meeting, the patients submitted completed digital reflection sheets and a nurse provided an asynchronous response in writing. The authors concluded that the intervention might be conducive to support the self-management of diabetes but that in-person consultations may be necessary to achieve the full potential of GSD [29]. eHealth intervention based on the GSD influenced the nurse-patient relationship by facilitating reciprocal understanding and flexibility [30], whereas GSD without face-to-face encounters reduced participant motivation to engage in the intervention [28]. Asynchronous writing, which may hamper the opportunities that nurses have to use basic and advanced communication skills, may not be adequate to replace the communication skills currently used in clinical practice [31]. This is in keeping with our findings, which show that the nurses were concerned about the deterioration of the nurse-patient relationship if videos hindered the interpretation of body language or if the conversations moved the focus to a shared screen. However, the nurses in our study found that they were able to communicate adequately and establish the type of relationship that was important for person-centered care during video conversations. The Australian web-based GSD program targeting young adults with type 1 diabetes, which used smartphones on Apple and Android platforms and Zoom (Zoom Video Communications) [32], found that the program not only promoted reflection and solutions, in addition to facilitating young adults’ journey based on their needs and goals, but also changed the way HCPs and patients collaborated. Issues regarding a lack of mobile responsiveness and saving material, which is also a factor in our study, required improvement [32]. To our knowledge, Denmark is the first country to host a web version of GSD on a national health portal, which increases the possibility of scaling up the intervention for wide implementation.

### Strengths and Limitations

Various factors may have affected our data, analyses, and findings. First, the oncology patient group provided feedback on the newly developed version of the DA-GSD and the endometriosis patient group did so on a more mature version, which may have negatively affected the experience of the former group. Second, the patients with cancer were older and perhaps more cognitively affected because of their diagnosis and treatment than the patients with endometriosis, who were younger, of childbearing age, and especially challenged by pain, making the comparison of their technological skills difficult. Third, in March 2020, the COVID-19 pandemic affected treatments, priorities, and the development of technology in the Danish health care system, although video consultations were quickly implemented to reduce the spread of COVID-19. These circumstances likely accelerated the implementation of the DA-GSD video feature. Fourth, the prospective use of a systematic evaluation tool, such as nonadoption, abandonment, scale-up, spread, and sustainability-complexity assessment tool, from the beginning of the research project may have amplified the attention given to the complex social and political aspects of the implementation process [34]. Fifth, only women participated in evaluating DA-GSD, which means that our findings are not directly applicable to men.

One of the strengths of this study is that it examines the perspectives of both nurses and patients regarding DA-GSD. Another strength is the longer-time perspective of 5 years. Moreover, several researchers participated in the analysis and interpretation of results, which enhances their reliability.

### Conclusions

In this study, the nurses experienced more barriers to DA-GSD than the patients. The acceptance of DA-GSD increased over time among the nurses once DA-GSD’s functionality improved, they had received guidance, and had had their own positive experiences, combined with the fact that the patients found DA-GSD useful and beneficial. Our findings emphasize the importance of focusing on supporting and teaching nurses if new technologies are to be implemented successfully.

## Abbreviations

DA-GSD: digitally assisted guided self-determination
DNHP: Danish National Health Portal
GSD: guided self-determination
HCP: health care professional
